# Impact of Sacubitril/Valsartan in Improving Home Time for Patients With Heart Failure

**DOI:** 10.7759/cureus.73175

**Published:** 2024-11-06

**Authors:** Jagdish Hiremath, SN Routray, Prakash Hazra, Dheeraj Gandotra, C K Ponde, Bijay P Pandey, Govindan Unni, Rajat Sharma, Natarajan Shivkadaksham, Sunil Sathe, Chandrashekhar Makhale, Nikhil Kumar

**Affiliations:** 1 Cardiology, Ruby Hall Clinic, Pune, IND; 2 Cardiology, SCB (Srirama Chandra Bhanja) Medical College and Hospital, Cuttack, IND; 3 Cardiology, Manipal Hospitals, Dhakuria, IND; 4 Cardiology, Interventional Cardiology and Heart Failure Program, BLK-Max Super Specialty Hospital, New Delhi, IND; 5 Cardiology, P. D. Hinduja National Hospital, Mumbai, IND; 6 Interventional Cardiology, Narayana Superspeciality Hospital, Howrah, IND; 7 Cardiology, Jubilee Mission Medical College and Research Institute, Thrissur, IND; 8 Heart Rhythm and Pacemaker Division, Interventional Cardiology, Fortis Hospital, Mohali, IND; 9 Interventional Cardiology, Siva Cardio Care, Chennai, IND; 10 Cardiology, Cardiac Care and Counselling Center, Pune, IND; 11 Cardiology, Fortis Hospital, Gurgaon, IND

**Keywords:** arni, heart failure, home time, hospitalizations, quality of life, reduced ejection fraction, sacubitril/valsartan

## Abstract

Home time, defined as time spent by the patient alive and out of any healthcare institution, is an important patient-centric outcome for patients with cardiovascular disease. Home time is recognized as a crucial measure of recovery post cardiovascular events but has not been extensively studied in heart failure (HF) patients, especially in India. HF in India is rapidly growing at an epidemic scale and hence the focus on improving home time in HF patients highlights the need for precise, patient-centered care strategies. Current literature lacks detailed descriptions of hospital-level patterns and predictors of home time in contemporary HF populations, which hinders tailored approaches to optimize outcomes like functional status and health-related quality of life along with reduced hospitalization and mortality risks. Literature is abundant with clinical evidence on the benefits of guideline-directed medical therapy (GDMT), especially angiotensin receptor neprilysin inhibitor (ARNI) therapy, in HF management. All major guidelines highly recommend its initiation for reducing morbidity and mortality in patients with chronic symptomatic HF with reduced ejection fraction. Studies indicate that sacubitril/valsartan, the first in class of ARNI, improves the quality of life and functional outcomes, along with reduced HF-related hospitalizations and cardiovascular deaths. Its unique mechanism of action, combining neprilysin inhibition and angiotensin receptor blockade, targets multiple pathways of HF pathophysiology, leading to improved cardiac function and remodeling. These benefits are pivotal in supporting patients' ability to maintain an active lifestyle outside of healthcare settings. Despite its demonstrated benefits, sacubitril/valsartan is underutilized. Integrating sacubitril/valsartan more optimally into clinical practice could significantly alleviate the overall burden of HF by addressing key determinants of home time and improving patient outcomes post discharge.

## Introduction and background

Heart failure (HF) epidemiology remains a pressing issue globally, affecting approximately 60 million people around the globe [1]. In India, an estimated 1% of the total population (8-10 million), are affected by HF, with an annual mortality rate estimated between 0.1 and 0.16 million individuals [2]. The incidence rate for hospitalization remains high with nearly one in four patients being hospitalized within 30 days after discharge and one in two patients being rehospitalized within six months. The mortality rate within one year and five years post discharge is 10% and 50%, respectively [3].

Available evidence indicates that despite conventional and novel management strategies, the steady or decreasing incidence of HF is inversely proportional to the mortality and hospitalization rate [3]. These concerning patterns highlight the complex nature of HF, the inadequate understanding of its diverse manifestations, and the challenges involved in managing it as a chronic disease. Currently, survivors of cardiovascular events are actively seeking effective treatments and care strategies to improve outcomes that matter most to them, such as functional status, quality of life (QoL), and the duration spent outside healthcare facilities, known as home time [4,5]. Home time is validated as a meaningful indicator of functional recovery post any cardiovascular event [5-7]. Despite the acknowledged importance of home time, current literature lacks descriptions of hospital-level patterns and predictors of this outcome in contemporary HF populations. One of the management strategies that has been shown to improve functional outcomes and optimally address the determinants of home time is therapy with sacubitril/valsartan (SV) [8,9]. SV has shown a considerable reduction in risk of hospitalization and cardiovascular death alongside improvements in QoL expressed as improvement in Kansas City Cardiomyopathy Questionnaire (KCCQ) clinical summary scores and New York Heart Association (NYHA) class [8,9]. Despite clear evidence demonstrating its superiority over standard treatments, only a small proportion of patients receive SV [10]. Barriers to optimal initiation of SV may include healthcare provider’s unfamiliarity with its clinical benefits, physician inertia to initiate, patients’ inertia to adoption, low awareness for HF management, loss to follow up, and concerns about its adverse effects among others [10,11]. Optimal integration of SV into clinical practice has the potential to significantly address all the determinants of home time and alleviate the overall burden of HF [8,9]. With this background, a group of cardiologists from India gathered together in an expert opinion forum meeting. They reviewed available literature evidence and provided individual insights based on their experience in managing HF with a primary focus on the role of SV in improving home time in HF patients.

## Review

Impact of heart failure on home time

**Figure 1:**
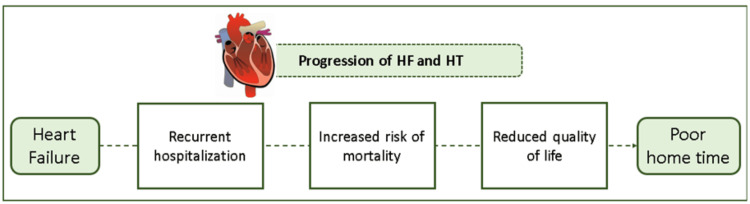
The impact of HF progression on HT. HF, heart failure; HT, home time. Image credits: Dr. Jagdish Hiremath. Adapted from Roger VL [4].

The concept of home time, defined as the time a patient spends alive and out of a healthcare institution, has been studied in ischemic stroke populations where it was shown to be a robust and easily measured patient-centered outcome [5]. Description of home time in HF may better communicate associated morbidity and mortality, including the burden of repeated hospitalizations, and time spent in rehabilitation and nursing facilities. Moreover, from a clinical trial perspective, home time could represent a novel trial endpoint that intrinsically reflects mortality and hospitalization events, but with a patient-centered approach [6]. The impact of HF progression on home time has been illustrated in Figure 1.

Sacubitril/valsartan in the management of heart failure and home time

**Figure 2:**
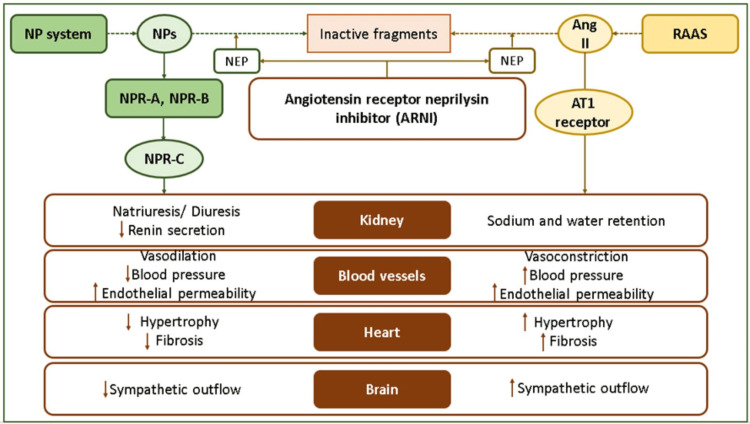
Mechanism of action of sacubitril/valsartan (ARNI). Ang II, angiotensin II; ARNI, angiotensin receptor neprilysin inhibitor; AT1, angiotensin type 1 receptor; CV, cardiovascular; NEP, neprilysin; NP, natriuretic peptide; NPR, natriuretic peptide receptor; RAAS, renin-angiotensin-aldosterone system. Image credits: Dr. Jagdish Hiremath. Adapted from McDonagh et al. [16].

HF pathophysiology involves renin-angiotensin-aldosterone system (RAAS) activation, leading to vasoconstriction, hypertension, increased aldosterone, sympathetic tone, and cardiac remodeling. Counter regulatory mechanism mediated by the natriuretic peptide (NP) system, which inhibits RAAS and promotes vasodilation, diuresis, and natriuresis, is diminished by the secretion of the enzyme neprilysin that degrades the NP. SV is a first-in-class angiotensin receptor-neprilysin inhibitor (ARNI) that combines a neprilysin inhibitor (sacubitril) to prolong NP effects and an angiotensin receptor blocker (ARB; valsartan) to inhibit RAAS [12-15]. The mechanism of action of SV has been illustrated in Figure 2.

**Figure 3:**
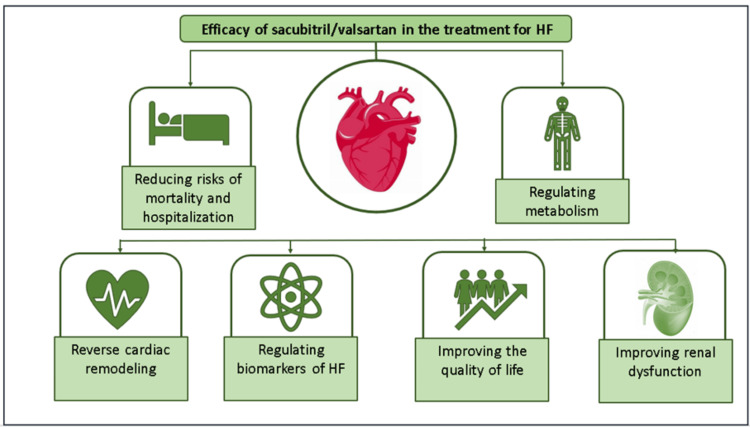
Efficacy of sacubitril/valsartan in the management of heart failure (HF). Image credits: Dr. Jagdish Hiremath. Adapted from Heidenreich et al. [17] and Zhang et al. [18].

SV has demonstrated significant efficacy in reducing both mortality and lowering the incidence of hospitalizations due to HF exacerbations and cardiovascular events among patients with heart failure with reduced ejection fraction (HFrEF) [8,12,13]. Thus, SV helps increase the amount of time patients can spend at home rather than in healthcare facilities or undergoing rehabilitation. This improvement in home time reflects better overall health stability and reduced reliance on acute medical care, contributing to improved QoL for patients. In addition to this, SV is associated with improvements in symptoms, heart function, and cardiac remodeling, which further support its role in enhancing the patient’s ability to maintain an active lifestyle at home [14-16]. The role of SV in the management of HF has been illustrated in Figure 3.

Major clinical guidelines for the management of HF strongly recommend SV alongside guideline-directed medical therapy (GDMT) such as beta-blockers (metoprolol succinate extended release, bisoprolol, and carvedilol), sodium-glucose cotransporter 2 inhibitors (SGLT2i) (dapagliflozin or empagliflozin), and mineralocorticoid receptor antagonist (MRA) (spironolactone, eplerenone) [16,17].

Clinical evidence of sacubitril/valsartan in improving home time

**Figure 4:**
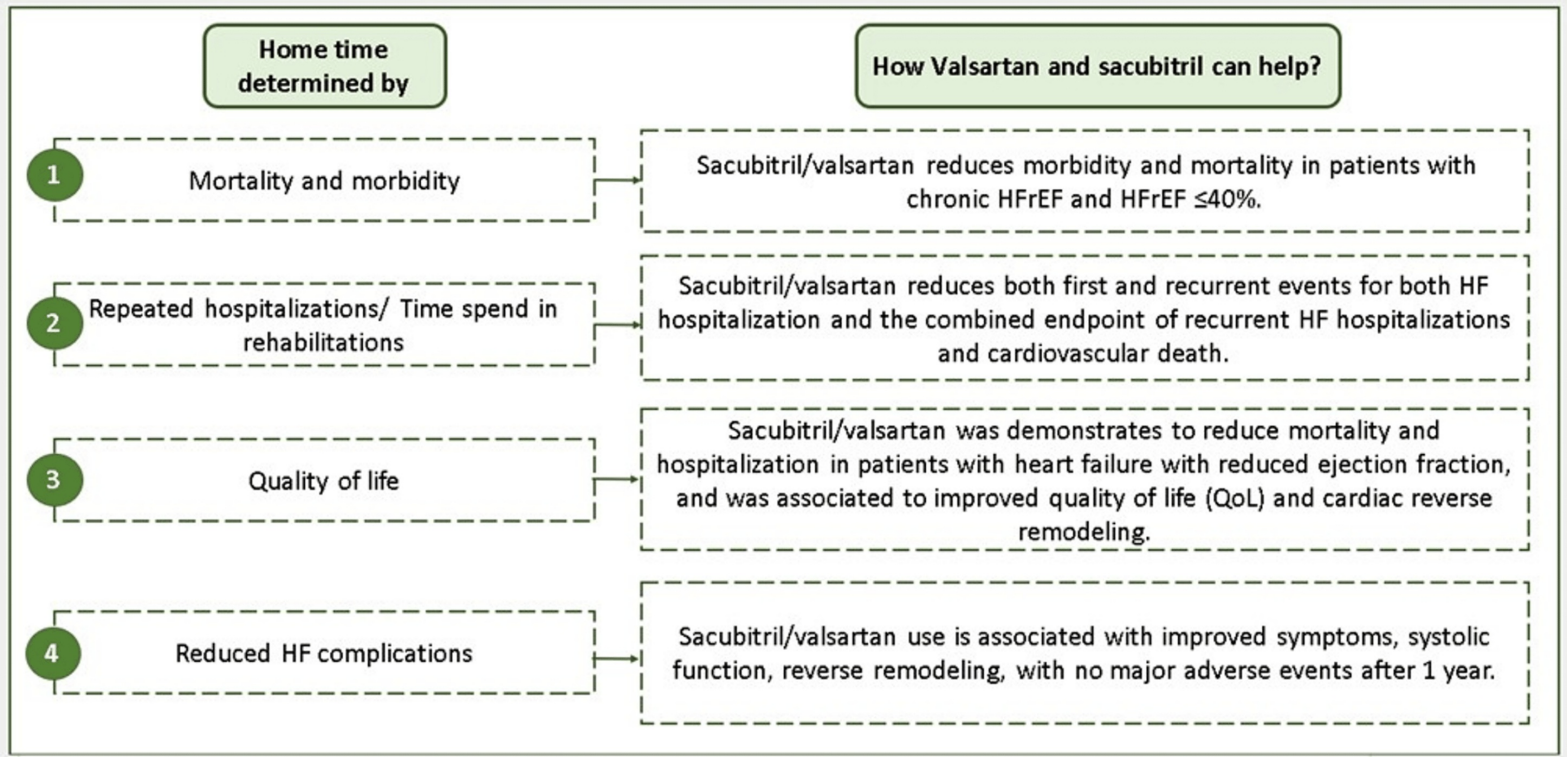
Role of sacubitril/valsartan in improving home time. HF, heart failure; HFrEF, heart failure with reduced ejection fraction. Image credits: Dr. Jagdish Hiremath. Adapted from Heidenreich et al. [17], Velazquez et al. [21], Balmforth et al. [22], and Jhund et al. [23].

The effectiveness of SV in managing HF can be majorly assessed across seven main aspects: lowering mortality and hospitalization risks, reversing cardiac remodeling, regulating HF biomarkers, enhancing QoL, improving renal function, and regulating metabolism in HF patients [18-20]. These are all the determinants influencing the home time of the patients and improvement in these parameters can directly extend their home time. The role of SV in improving home time has been illustrated in Figure 4.

Role of Sacubitril Valsartan in Risk Reduction of Heart Failure Hospitalization and Mortality

A primary goal of treating patients with HFrEF is to reduce the occurrence of frequent hospitalizations for worsening HF [16]. The transition from hospital to home represents a vulnerable period due to the progressive complexity of HF. Patients admitted for HF face a high event rate, with mortality ranging from 10% to 15% and readmission rates between 30% and 40% within six months post discharge. Despite advancements in HF treatments that have reduced mortality rates, these developments have not culminated in lower hospitalization rates [19].

Hospitalization is a key determinant of home time in patients with HF. SV has demonstrated efficacy in reducing the risk of hospitalization due to HF in patients with chronic HFrEF and in patients hospitalized due to acute decompensation [20,21]. The PARADIGM-HF trial, comparing SV with angiotensin-converting enzyme inhibitor (ACEi) like enalapril, demonstrated a 20% relative risk reduction (RRR) in the primary composite endpoint of cardiovascular death or HF hospitalization. It also showed a 20% reduction in cardiovascular death, a 21% reduction in first HF hospitalization, and a 16% reduction in all-cause mortality over and above an ACEi, over a median follow-up of 27 months [13]. This benefit remained consistent regardless of the underlying etiology of HF or the patient's age [22-24]. The PIONEER-HF trial further supported these findings by demonstrating a 44% risk reduction of HF rehospitalization with that SV compared to ACEi, in patients with acute decompensated HF, over an eight-week follow-up period [21].

Role of Sacubitril/Valsartan in Heart Failure Across Ejection Fraction Spectrum

There is a consistent trend that the worse the left ventricular systolic function, the greater the likelihood of benefit from standard HF treatment. This was true for ACEi, beta-blockers, and MRA, and now seems to be true for ARNI as well. The benefits of SV compared to a RAAS inhibitor alone differ depending on left ventricular ejection fraction (LVEF) [25,26]. The PARADIGM-HF study established the superiority of SV over ACEi in the HFrEF population wherein the LVEF was ≤ 40% [13]. Hence the major guidelines recommend ARNI as the first-line drug in HFrEF management with class I recommendation [16]. However, the results are not consistent as LVEF increases to normal and above-normal values. In the PARAGON-HF trial, treatment with SV in HF patients with LVEF ≥ 45% showed a numerical reduction in the composite outcome of total HF hospitalizations and cardiovascular death compared with valsartan, but the result was not statistically significant [27]. The pooled data from PARADIGM-HF & PARAGON-HF trials (n = 13195) showed SV to be superior to RAAS inhibitor (ACEi/ARB) with 16% RRR for first cardiovascular death or HF hospitalization, 16% RRR for cardiovascular death, 16% RRR for HF hospitalization, and 12% RRR for all-cause mortality [28]. Thus, the beneficial effect of SV was impacted by LVEF, and the benefit appeared to be present for individuals with LVEF below the normal range. A meta-analysis study of evidence with a total of 1937 patients of heart failure with mildly reduced ejection fraction (HFmrEF) and LVEF of 41-49% suggested SV may be an effective strategy to reduce HF hospitalization (RR: 0.54, (95%CI: 0.43-0.68), P < 0.005). [29] The PARAGLIDE-HF study, which assessed the effects of SV in HFmrEF patients following a recent worsening HF event, further supported its beneficial effects compared to ARB [30]. The pooled analysis of all the patients from PARAGLIDE-HF and PARAGON-HF (n = 5262) showed a significant reduction of the total worsening HF events and cardiovascular death by 14% and this clinical benefit was observed as early as within nine days of treatment initiation in patients with LVEF up to 60% [31]. All this evidence repeatedly bolsters the clinical benefits of SV in HF patients across the ejection fraction spectrum. The guideline recommendations for the use of SV in HF have been enlisted in Table 1.

**Table 1:** Guideline recommendations on the use of sacubitril/valsartan in HFrEF. ACC, American College of Cardiology; ACEi, angiotensin-converting enzyme inhibitor; AHA, American Heart Association; ARB, angiotensin receptor blocker; COR, class of recommendation; ESC, European Society of Cardiology; HF, heart failure; HFrEF, heart failure with reduced ejection fraction; LOE, level of evidence; NYHA, New York Heart Association.

Guideline	Recommendations	COR	LOE
ESC 2021 [16]	Sacubitril/valsartan is recommended as a replacement for ACEi in patients with HFrEF to reduce the risk of HF hospitalization and death.	1	B
ACC/AHA 2022 [17]	In patients with HFrEF and NYHA class II to III symptoms, the use of sacubitril/valsartan is recommended to reduce morbidity and mortality.	1	A
	In patients with chronic symptomatic HFrEF NYHA class II or III who tolerate an ACEi or ARB, replacement by sacubitril/valsartan is recommended to further reduce morbidity and mortality.	1	B

Role of Sacubitril/Valsartan in the Improvement of Cardiac Function

In the PROVE-HF trial involving 794 HFrEF patients, SV increased LVEF by 9.4% (P < 0.001) and decreased left ventricular end-diastolic volume index by 12.25 mL/m2 (P < 0.001), left ventricular end-systolic volume index by 15.29 mL/m2 (P < 0.001), left atrial volume index by 7.57 mL/m2 (P < 0.001), and the E/e’ ratio by 1.3 (P < 0.001) after 12 months of treatment [32]. Another randomized controlled trial indicated a significantly greater reduction in effective regurgitant orifice area among HFrEF patients with functional mitral regurgitation treated with SV compared to those treated with valsartan alone (-0.058 ± 0.095 vs. -0.01 ± 0.105 cm2; P = 0.032) [33]. These findings demonstrate significant improvements in cardiac structure and function among HFrEF patients treated with SV. A retrospective cohort study revealed that SV treatment for 12 months significantly enhanced the reservoir function of atrial chambers (P < 0.001), as assessed by peak atrial longitudinal strain, compared to treatment with RAAS inhibitors in patients with HF. Additionally, improved reservoir function of the atrial chambers was linked to a reduced risk of atrial fibrillation (AF) recurrence in this study [34,35].

Role of Sacubitril/Valsartan in Functional Capacity Assessment in Heart Failure

HF is characterized by reduced exercise capacity and marked by symptoms such as dyspnea, fatigue, and edema. As the condition advances, patients often experience a decline in physical activity, exacerbating their exercise intolerance. This functional decline can impair their ability to perform daily tasks and may even lead to work limitations or loss. Furthermore, HF patients commonly face challenges in managing daily activities and have diminished QoL [35-37].

Six-minute walk test: The six-minute walk test (6MWT) is a commonly used exercise assessment to evaluate the physical functional capacity of patients with HF. It is a simple tool that can help predict morbidity and mortality [38]. It is known that a distance of less than 350 meters during the 6MWT is associated with increased mortality in HF patients [39]. In the BIOSTAT-CHF study, walking 240 meters or less at the baseline assessment was shown to be more predictive of mortality, than age (>75 years), diabetes, chronic renal failure, or previous stroke. A decrease in 6MWT by 50 meters increased the risk of composite mortality and hospitalization due to HF by 8% and the risk of mortality by 14% [38]. The available evidence points toward the fact that SV can potentially improve the 6MWT distance and exercise tolerance in patients with HFrEF. The OUTSTEP-HF trial, comparing the immediate impacts of SV versus ACEi on daily physical activity in patients with chronic HFrEF, demonstrated that SV improved 6MWT by 35 meters after 12 weeks. Nearly half of SV-treated patients improved their 6MWT distance by at least 30 meters [39]. Another study by Sgorbini et al. observed that the average distance walked increased from 129 meters to 436 meters with one month of SV treatment [40].

New York Heart Association classification: The NYHA classification system for HF involves a clinician's subjective assessment aimed at evaluating the functional capacity and symptoms of individuals diagnosed with symptomatic HF. It serves as an independent predictor of mortality and is routinely utilized in clinical settings to determine appropriate therapeutic interventions for these patients. The NYHA class is assessed by the clinician at baseline and at follow-up periods to determine changes in functional capacity and symptoms of HF [17]. In a comparative analysis of pre-post treatment with SV in HFrEF patients (n = 105), a notable improvement in NYHA class was observed within 30 days. The proportion of patients in NYHA class I increased from 8.6% to 32.4%, while the proportion in NYHA class II & class III changed from 73.3% & 18.1% to 58.1 & 9.5%, respectively. About 34.3% of patients demonstrated advancement of at least one NYHA class category [41]. A real-world evidence study with SV in HFrEF patients (n = 90) corroborated similar improvement in the NYHA class. At baseline, all patients belonged to NYHA II (52%) & NYHA III (48%), and at six months post SV treatment, the proportions changed to 13%, 78%, and 9% in NYHA class I, II, and III, respectively [42]. Another observational study, the Real-World Evidence study (REAL.IT), observed that 37.5% of patients showed improvement in at least one NYHA class and the proportion of patients in NYHA class III reduced from 36.1% at baseline to 16.7% after one year of follow-up [43].

Role of Sacubitril/Valsartan in Improving Quality of Life in Heart Failure

The primary goals of treating HF include minimizing disease progression (death, hospitalization) and optimizing patients’ health status and their symptoms, function, and QoL [16]. As the disease progresses, cardiac function falls and the risk of recurrent hospitalization and mortality increases, thus affecting the patient's QoL and home time. The worsening of HF condition is marked by the increase in the NYHA class category and limitation to physical activity [4,6]. While the NYHA assessment is simple, it has several limitations owing to its subjective nature. Several questionnaire-based methods of assessment of QoL have been developed and adopted in clinical trials and the KCCQ is one of the most commonly used methods [44-46]. The KCCQ measures symptoms, physical and social limitations, and QoL in patients with HF and is a more standardized, quantification tool to identify changes in patients’ health status. The KCCQ effectively and reliably measures the impact of HF on the lives of patients through consistent questioning over time and demonstrating a strong correlation with clinical events [47].

Treatment with SV not only improves morbidity and mortality but also reduces physical and social activity limitations and improves emotional well-being [14,44]. Noteworthy improvements were observed with household chores and sexual relationships within eight months and persisted up to 36 months with SV treatment [44]. Sub-analysis of PARADIGM-HF showed that patients who received SV exhibited enhancements in the KCCQ clinical summary score (+0.64 compared to −0.29; P = 0.008) and KCCQ overall summary score (+1.13 versus −0.14; P < 0.001) relative to those who received ACEi at eight months. A notably reduced percentage of SV patients experienced deterioration (≥5 points decrease) in both KCCQ scores (27% versus 31%; P = 0.01) [14]. Switching from ACEi to SV resulted in notable improvement in various measures of depression and anxiety among patients with HFrEF [47]. Regular monitoring of QoL using questionnaire-based tools like the KCCQ allows healthcare providers to track these improvements over time, ensuring that treatment adjustments can be made promptly to optimize outcomes. The Indian Council of Medical Research (ICMR) has developed an Indian HF-specific QoL questionnaire that is a valid, reliable, sensitive, and responsive tool and can be integrated into routine clinical practice for assessing QoL among Indian HF patients.

Dose of sacubitril/valsartan for improving home time

SV is recommended as a replacement for ACEi in all eligible HFrEF patients by the clinical guidelines for HF management [16,17]. The recommended initiation dose is 100 mg twice per day (BID) in patients with well-tolerated high doses of ACEi. Initiation with a low dose of 50 mg BID is recommended in patients who have not received ACEi/ARB or administered low doses of these drugs, as well as individuals with severe renal dysfunction. The decision on further dose management should be followed by an evaluation of patients’ hemodynamic stability, renal function, and potassium levels. It is recommended to gradually uptitrate the dose of SV to the target dose of 200 mg BID or to the maximal dose tolerated by the patient within two to four weeks after initiation [48]. A study with 794 HFrEF patients evaluated the efficacy of differential dosing of SV at an average daily dose of 112 mg (low dose), 342 mg (moderate dose), and 379 mg (high dose) through a study period of 12 months. The study demonstrated similar improvement in cardiac function and health status across all doses of SV [49]. A similar observation was noted in a real-world study wherein clinical and functional improvement in HFrEF patients was achieved with SV treatment with a higher dose resulting in a greater magnitude of benefit [50]. The recommended dosage for SV treatment in HFrEF patients has been enlisted in Table 2.

**Table 2:** Recommended initiation doses of sacubitril/valsartan among HFrEF patients. ACEi, angiotensin-converting enzyme inhibitor; ARB, angiotensin receptor blocker; eGFR, estimated glomerular filtration rate; HFrEF, heart failure with reduced ejection fraction. Adapted from Gori et al. [48].

Type of patient	Recommended initiation dose of sacubitril/valsartan
Patients currently on low-dose ACEi or those who have not previously been treated with ACEi	Initiate sacubitril/valsartan therapy with a dose of 50 mg (24 mg sacubitril/26 mg valsartan) orally twice daily.
Patients currently on moderate to high doses of ACEi	The recommended initiation dose of sacubitril/valsartan is 100 mg (49 mg sacubitril/51 mg valsartan) orally twice daily.
Patients with mild renal impairment (eGFR: 60-90 ml/min/1.73 m2)	No dose adjustment of sacubitril/valsartan is required.
Patients with moderate renal impairment (eGFR: 30-60 ml/min/1.73 m2)	An initial dose of 50 mg (24 mg sacubitril/26 mg valsartan) orally twice daily should be considered.
Patients with severe renal dysfunction (eGFR < 30 ml/min/1.73 m2)	Sacubitril/valsartan should be used with caution and a starting dose of 50 mg (24 mg sacubitril/26 mg valsartan) twice daily may be considered.
Patients with systolic blood pressure ≥ 110 mm Hg	No dose adjustment is required.
Patients with systolic blood pressure < 100 mm Hg	Sacubitril/valsartan should be used with caution and a starting dose of 50 mg (24 mg sacubitril/26 mg valsartan) orally twice daily should be considered.
Patients with serum potassium levels > 5.4 mmol	Sacubitril/valsartan treatment should not be initiated.

Underutilization of sacubitril/valsartan in India

The PARADIGM-HF trial in 2014 established SV as a successful treatment for HF [13]. Recent studies further endorse broadening its application, and major clinical guidelines strongly recommend its use as a first-line drug in HFrEF management [16,17]. Despite the positive clinical evidence and clear clinical guideline recommendations, there is marked underutilization of this drug as evident from the national and international HF registry data [51-54]. The adoption of SV in HF management faces a few challenges that impact its widespread adoption. Financial constraints pose a significant hurdle, as the cost of SV therapy may limit its accessibility, particularly in resource-limited settings. Concerns over potential adverse effects such as hypotension and renal impairment further complicate initiation and titration decisions among physicians. Moreover, there is a notable lack of awareness among healthcare providers regarding its efficacy and safety, particularly in patients with low blood pressure or renal dysfunction. Patient adherence is also hindered by limited awareness of its benefits and potential side effects, underscoring the need for comprehensive education efforts. Transitioning from established ACEi therapies can be challenging due to conventional clinical practices and concerns over patient response [55]. However, continuous education initiatives targeting both healthcare professionals and patients, along with efforts to improve affordability and streamline access, may be crucial in overcoming these barriers. The evidence-based expert opinions on the role of SV in HF and improving home time have been enlisted in Table 3.

**Table 3:** Expert opinions on the role of sacubitril/valsartan in HF and home time improvement. 6MWT, six-minute walk test; ACEi, angiotensin-converting enzyme inhibition; ARB, angiotensin II receptor blocker; HF, heart failure; EF, ejection fraction; HF, heart failure; HFrEF, heart failure with reduced ejection fraction; KCCQ, Kansas City Cardiomyopathy Questionnaire; LVEF, left ventricular ejection fraction; NYHA, New York Heart Association; PARADIGM-HF, Prospective Comparison of Angiotensin Receptor-Neprilysin Inhibition with Angiotensin-Converting Enzyme Inhibition in Heart Failure; PIONEER-HF, Comparison of Sacubitril–Valsartan versus Enalapril on Effect on NT-proBNP in Patients Stabilized from an Acute Heart Failure Episode; PROVE-HF, Prospective Study of Biomarkers, Symptom Improvement, and Ventricular Remodeling During Sacubitril/Valsartan Therapy for Heart Failure; QoL, quality of life; RAAS, renin-angiotensin-aldosterone system.

Parameter	Expert opinion
Importance of home time in HF	Home time is a critical measure in HF management aimed at improving quality of life alongside reducing hospitalizations and mortality rates. Maximizing home time in HF aims to improve quality of life and functional capacity through enhanced symptom management, optimized medication adherence, tailored care plans, and psycho-social support.
Role of sacubitril/valsartan in improving home time	Sacubitril/valsartan has demonstrated a significant reduction in hospitalizations for patients with HF, allowing them more time at home and in their communities, which enhances independence, comfort, and overall well-being. This increased home time fosters engagement in social activities and strengthens social connections, thereby improving QoL. The improved cardiac function resulting from sacubitril/valsartan therapy enhances the patient’s ability to perform daily tasks and participate in physical activities, alleviating symptoms like fatigue and shortness of breath.
Role of sacubitril/valsartan in risk reduction of heart failure hospitalization and mortality	Sacubitril/valsartan reduces the risk of cardiovascular death and hospitalization due to heart failure, offering a substantial survival advantage compared to conventional RAAS inhibitors like ACEi or ARB. The role of sacubitril/valsartan in the reduction of the risk of HF hospitalization and mortality among HFrEF patients has been well established with large trials like PARADIGM-HF and PIONEER-HF. Major clinical guidelines for the management of HF strongly recommend the initiation of sacubitril/valsartan in all eligible HFrEF patients.
Role of sacubitril/valsartan across EF spectrum	Sacubitril/valsartan provides significant benefits in HFrEF and should be considered for initiation in all eligible patients The beneficial effects of sacubitril/valsartan vary with LVEF, and benefits appeared to be present for individuals with LVEF below the normal range.
Role of sacubitril/valsartan in the improvement of cardiac function	Sacubitril/valsartan operates via dual mechanisms involving inhibiting the RAAS and augmenting natriuretic peptides through neprilysin enzyme inhibition, thereby fostering improved ventricular remodeling and function. The PROVE-HF trial demonstrated the improvement in cardiac functions of ejection fraction and other echocardiographic parameters with 12 months of treatment with sacubitril/valsartan. Initiation of sacubitril/valsartan can yield improvements in cardiac function as early as one month after treatment initiation, with typical enhancements in LVEF and reductions in left atrial volume index within three months.
Significance of 6MWT assessment in HF	Routine use of the 6MWT is recommended in HF clinics due to its ability to provide objective evidence of improvement and enhance patient confidence. 6MWT is still underutilized in clinical practice, with few clinicians routinely incorporating it into patient assessments. Logistical constraints may limit the feasibility of the 6MWT in outpatient settings.
Role of sacubitril/valsartan in improvement in 6MWT	There is marked improvement in the functional capacity and definitive improvement observed in 6MWT with 3-6 months of sacubitril/valsartan therapy.
Significance of NYHA class assessment in HF	Assessment of NYHA class through patient history and clinical evaluation is the simplest way to assess exercise tolerance and physical limitations in HF patients. Clinicians commonly assess improvement in NYHA class using simple questionnaires that evaluate functional capacity, such as walking ability and exertional tolerance. One of the important treatment objectives should be to achieve at least one class improvement in NYHA functional classification (e.g., from NYHA III to II).
Role of sacubitril valsartan in improving NYHA class in HF	Significant improvement in NYHA class is typically seen within 3-6 months of sacubitril/valsartan initiation.
Significance of quality-of-life assessment in HF	One of the primary goals of treating HF is optimizing patients’ health status—their symptoms, function, and quality of life. Questionnaire-based evaluation of QoL (like KCCQ) can effectively and reliably measure the QoL of HF patients through consistent questioning over time. The Indian HF QoL questionnaire has been developed as a valid, reliable, sensitive, and responsive tool for assessing QoL among Indian HF patients.
Role of sacubitril/valsartan in improving QoL	Treatment with sacubitril/valsartan reduces physical and social activity limitations and improves emotional well-being. Noteworthy improvements were observed with household chores and sexual relationships. Sacubitril/valsartan treatment leads to improvement in various measures of depression and anxiety.

## Conclusions

SV represents a paramount advancement in managing HF, particularly in enhancing home time for HFrEF patients. By significantly reducing hospitalizations due to HF exacerbations and cardiovascular events, SV therapy allows patients to spend more meaningful time at home with their families and communities. This improvement in home time not only reflects better health stability and reduced reliance on acute medical care but also translates into enhanced QoL and functional status. Despite these beneficial outcomes, challenges remain in the widespread adoption of SV in clinical practice. Addressing barriers such as healthcare provider inertia and safety concerns is crucial to optimizing the integration of SV into HF management protocols. Future research should continue to explore the impact on home time and refine patient-centered care strategies aimed at maximizing functional outcomes and reducing the overall burden of HF on individuals and healthcare systems.
